# Recent developments in the structural characterisation of the IR and IGF1R: implications for the design of IR–IGF1R hybrid receptor modulators†

**DOI:** 10.1039/d1md00300c

**Published:** 2022-02-21

**Authors:** Samuel J. Turvey, Martin J. McPhillie, Mark T. Kearney, Stephen P. Muench, Katie J. Simmons, Colin W. G. Fishwick

**Affiliations:** Leeds Institute for Cardiovascular and Metabolic Medicine, University of Leeds UK K.J.Simmons@leeds.ac.uk; School of Chemistry, University of Leeds UK C.W.G.Fishwick@leeds.ac.uk; School of Biomedical Sciences, Faculty of Biological Sciences & Astbury Centre, University of Leeds UK

## Abstract

The insulin receptor (IR) and insulin-like growth factor 1 receptor (IGF1R) are dimeric disulfide-linked receptor tyrosine kinases, whose actions regulate metabolic and mitogenic signalling pathways inside the cell. It is well documented that in tissues co-expressing the IR and IGF1R, their respective monomers can heterodimerise to form IR–IGF1R hybrid receptors. Increased populations of the IR–IGF1R hybrid receptors are associated with several disease states, including type 2 diabetes and cancer. Recently, progress in the structural biology of IR and IGF1R has given insights into their structure–function relationships and mechanism of action. However, challenges in isolating IR–IGF1R hybrid receptors mean that their structural properties remain relatively unexplored. This review discusses the advances in the structural understanding of the IR and IGF1R, and how these discoveries can inform the design of small-molecule modulators of the IR–IGF1R hybrid receptors to understand their role in cell biology.

## Abstract 2

This review discusses advances in the structural understanding of the insulin receptor (IR) and insulin-like growth factor 1 receptor (IGF1R) and how these discoveries can inform design of small-molecule modulators of the IR–IGF1R hybrid receptors.

## Graphical abstract

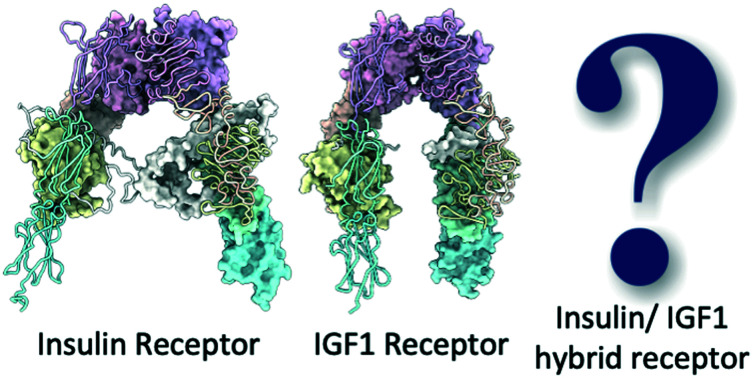

## Introduction

Over the past four decades changes in human lifestyle have contributed to an explosion of obesity^11,12^ and its frequent sequelae insulin resistant type 2 diabetes mellitus.^15^ A poorly understood hallmark of obesity and type 2 diabetes mellitus is disruption of insulin signalling,^17,18^ leading to dysregulation of cellular growth and nutrient handling.^20^ The insulin receptor (IR) acts as a conduit for insulin-encoded information, which is transferred *via* a complex intracellular signalling network including the critical signalling nodes phosphatidylinositol 3-kinase (PI3-K) and the serine/threonine kinase Akt, to regulate cell metabolism.^21^ During evolution the IR and insulin-like growth factor-1 receptor (IGF1R) diverged from a single receptor in invertebrates,^22,23^ into a more complex system in mammals.^24^ Stimulation of IR or IGF1R initiates phosphorylation of IR substrate (IRS) proteins at multiple tyrosine residues,^21^ phosphorylated IRS1 binds PI3-K initiating the conversion of the plasma lipid phosphatidylinositol 3,4-bisphosphate to phosphatidylinositol 3,4,5-trisphosphate (PIP3) which activates the multifunctional serine–threonine kinase Akt.^25^ In endothelial cells Akt activates the endothelial isoform of nitric oxide synthase (eNOS) by phosphorylation of serine 1177.^26,27^ In humans and other mammals despite high structural homology and activation of similar downstream pathways the biological processes regulated by insulin and IGF-1 are strikingly different.^28^ Consistent with this in endothelial cells Duncan and Sukumar *et al.* demonstrated that deletion of IR reduced,^29,30^ whereas deletion of IGF1R increased basal serine 1177 phosphorylated eNOS and insulin-mediated phosphorylation of serine 1177 on eNOS.^31^ They also showed that increasing IR in endothelial cells enhances insulin-mediated serine phosphorylation of Akt but blunts insulin-mediated serine 1177 phosphorylation of eNOS,^32^ whereas increased IGF1R reduces basal serine 1177 phosphorylated eNOS and insulin-mediated serine 1177 phosphorylation of eNOS.^33^

**Fig. 1:**
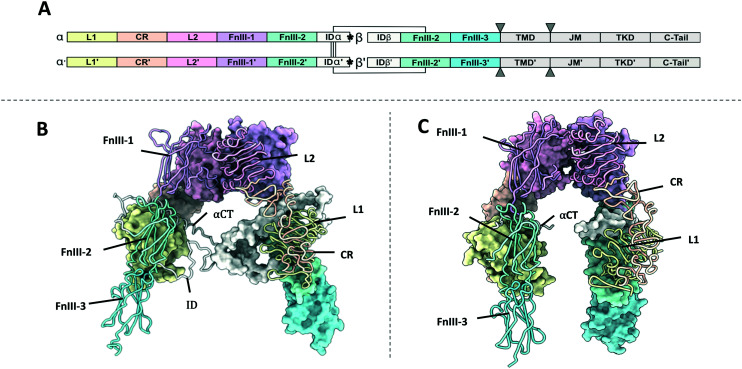
(A) Domain organisation of the IR and IGF1R. Monomers have been denoted as monomer and monomer' for clarity. The position of the αCT helix is denoted with asterixis (*). The position of the cell membrane has been indicated with triangles (▼); (B) XRCD apo-structure of the IR ectodomain (PDB: 4ZXB^2^). One monomer is shown with a space filling representation and the other with a ribbon representation; (C) XRCD apo-structure of the IGF1R ectodomain (PDB: 5U8R^3^). One monomer is shown with a space filling representation and the other with a ribbon representation. Note – the ID is not resolved in the IGF1R structure.

The insulin receptor (IR) and insulin-like growth factor receptor (IGF1R) are closely related multi-domain receptor tyrosine kinases (RTKs) that share *ca.* 70% sequence homology (Fig. 1A). The IR is activated through binding to insulin, whilst IGF1R is activated through binding to the hormones insulin-like growth factor (IGF) 1 and IGF2.^34^ Upon binding to their respective ligands, IR primarily regulates metabolic signalling through the PI3K/AKT pathway, whilst IGF1R elicits mitogenic effects through the Ras/ERK pathway. However, there is substantial crossover in the signalling of the receptors through the shared signalling nodes of the insulin receptor substrate 1, IRS2 and Shc. Similarly, IR and IGF1R can bind insulin, IGF1 and IGF2, albeit with affinities such that they only bind their own cognate ligands at physiological concentrations.^35–37^ Due to the array of signalling pathways regulated by the IR and IGF1R, aberrant signalling through these receptors is associated with several diseases. Specifically, dysfunctional insulin signalling is the primary driver of type 2 diabetes mellitus, whilst altered IGF1 signalling manifests in several forms of cancer due to its role modulating cell proliferation.^38^

IR and IGF1R are unique amongst RTK's in that they exist on the cell surface membrane as preformed dimers, contrasting with other RTK's that only dimerise upon ligand binding. This means that the receptors share a unique mode of action amongst RTK's; hormone binding elicits a largescale conformational change to activate the receptor. The structural basis of activation of the insulin family receptors is still not fully understood, although recent findings have significantly contributed towards providing a plausible activation mechanism of the receptors.^39^ Ligands binding to the extracellular portion (ectodomain) of the receptors affect *trans*-autophosphorylation of the intracellular kinase domains, which in turn promotes binding and phosphorylation of adaptor proteins to affect subsequent downstream signalling.

Due to their high homology, the IR and IGF1R can heterodimerise to form functional hybrid receptors in tissues in which they are co-expressed, consisting of an IR monomer and IGF1R monomer. Hybrid receptors bind IGF1 with *ca.* fifty times higher affinity than insulin and *ca.* ten times higher affinity than IGF2.^35–37^ The physiological role, signalling properties and mechanisms regulating the formation of IR–IGF1R hybrid receptors are currently poorly understood. However, it is believed that hybrid receptors confer insulin resistance by sequestering IR protein and reducing the available insulin binding sites on the cell surface.^40,41^ Similarly, increased numbers of hybrid receptors may increase the number of receptors binding IGF1 and IGF2, and therefore contribute to the signalling of these receptors in certain cancers.^42^ In skeletal muscle, fat and the heart, hybrid formation has been shown to exceed that of IGF1R and IR dimers.^43^ While the role of hybrids in human physiology is undefined there is a clear association with increased hybrids and situations of metabolic stress including: type 2 diabetes mellitus,^44,45^ obesity,^46^ hyperinsulinemia,^47^ insulin resistance^48^ and hyperglycaemia.^49^

Whilst there have been significant recent advances in the structural information available for the IR and IGF1R, limited structural information exists for hybrid receptors. Through a combination of X-ray crystallography diffraction (XRCD), cryo-electron microscopy (Cryo-EM) and nuclear magnetic resonance spectroscopy (NMR), three-dimensional high-resolution structures exist for almost the entirety of IR and IGF1R in their unbound (apo) and ligand bound conformations. However, due to difficulties in purifying and isolating hybrid receptors no-such structures exist for these receptors, which limits efforts to design specific hybrid modulators. This review aims to cover two areas: 1) recent advances in the structural biology of IR and IGF1R, and comment on their structural implications for hybrid receptors; 2) how a structural model of hybrid receptors can be used to inform the structure-based design of hybrid modulators.

## Biosynthesis of the IR, IGF1R and hybrid receptors

Both the IR and IGF1R are synthesised as single chain proreceptors, and subsequently undergo significant posttranslational processing including co-translational cleavage, glycosylation, and dimerization in the endoplasmic reticulum.^50,51^ It is thought that the stoichiometry of IR and IGF1R monomers present at this stage of receptor processing determines the relative ratios of IR, IGF1R and hybrid receptors. This is due to the observation that the relative proportions of homodimeric and heterodimeric receptors is proportional to their relative expression in a given tissue.^52^ Furthermore, increased expression of the IGF1R hemi-receptor drives the sequestration of the IR monomers into hybrids in both cultured fibroblasts^53^ and mice.^40,41^ Subsequently, the receptors are transported to the Golgi apparatus where they are cleaved into their characteristic α and β chains, and a none-globular insert domain added between the α and β subunits prior to membrane insertion.^51^

## Domain organisation of the IR and IGF1R

The IR and IGF1R show similar domain organisation (Fig. 1B and C, Table S1†). The extracellular portion of each receptor monomer contains two leucine rich domains (L1 and L2), a cysteine rich domain (CR), and three fibronectin type III domains (FnIII-1, FnIII-2, and FnIII-3). The FnIII-2 domain contains the insert domain (ID) spanning the α and β subunits. The ID contains a key ligand binding region at the C-terminal end of the α-subunit termed the α C-terminal helix (αCT). The remainder of the β subunit contains a single-pass helical transmembrane domain (TMD), and an intracellular tyrosine kinase domain (TKD) flanked by regulatory juxta membrane and C-tail regions. Three disulfide bonds linking the αβ monomers are contained in the ID, whilst the single disulfide linking the α and β subunits is in the FnIII-2 domain (Fig. 1A).

## Ectodomain structures

### Apo-structures of the receptors

Apo-structures for both the IR^2^ (PDB: 4ZXB, Fig. 1B) and IGF1R^3^ (PDB: 5U8R, Fig. 1C) ectodomain have been determined using X-ray crystallography to 3.3 Å and 3.0 Å respectively. Both show a characteristic inverted V-shape with each αβ monomer arranged in an antiparallel fashion. The first leg of the V is formed by the L1, CR and L2 domains, which pack against the second leg formed by the FnIII-1′, FnIII-2′ and FnIII-3′ domains (the apostrophe denoting the domains are from the alternate monomer). The L2 and FnIII-1 domains comprise the apex of the V. This arrangement places the FnIII-3 domains distal to the membrane. Major intermonomer interfaces occur between the L2 and FnIII-1′, as well as the L1 and FnIII-2′ domains. The structures determine that the αCT helix lies across the second β-sheet of the L1′, forming the major hormone binding epitope.

As IR and IGF1R show comparable topology in their ligand-free forms, it is likely that hybrid receptors will adopt a similar V-shaped structure in their apo-conformation. The IR and IGF1R apo-structures contrast in the relative alignments between the receptor domains, which results in distances of 115 Å and 67 Å between the relevant FnIII-3 and FnIII-3′ domains respectively (corresponding to the sites of membrane entry). However, it is difficult to say whether this represents a real difference between the receptors apo-conformations or arises from lack of membrane restraints and subsequent lattice confinement in the crystallographic structures. If this is physiologically relevant, it is likely that hybrid receptors would adopt domain conformations intermediate of those observed in IR and IGF1R. Additionally, the interfaces formed in the IR and IGF1R ectodomain apo-structures are conserved: the L1–FnIII-2 interface and the L2–FnIII-1 interfaces. Therefore, it is likely these are conserved in the hybrid receptors. This raises the possibility of identifying small molecules to disrupt these interfaces. If these act prior to disulfide linkage of the two monomers, it may prevent IR–IGF1R formation, and represent useful tool compounds for elucidating the signalling capabilities of these hybrid receptors.

### IR ligand bound structures

**Fig. 2:**
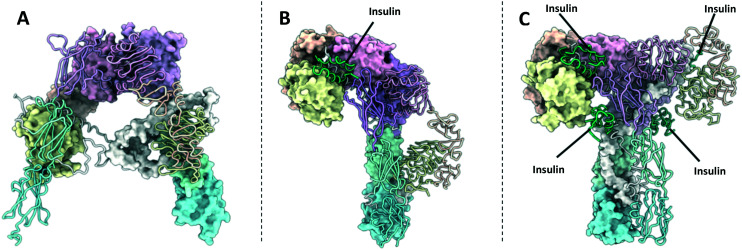
(A) XRCD structure of the apo-IR ectodomain (PDB: 4ZXB^2^); (B) Cryo-EM structure of the Γ-shaped IR ectodomain bound to a single insulin (PDB: 6HN4 and 6HN5 (ref. 4)); (C) Cryo-EM structure of the T-shaped IR ectodomain bound to four insulin (PDB: 6SOF^14^)). One monomer in each structure is depicted with a molecular surface and the other in cartoon format. Insulin is depicted in green.

**Fig. 3:**
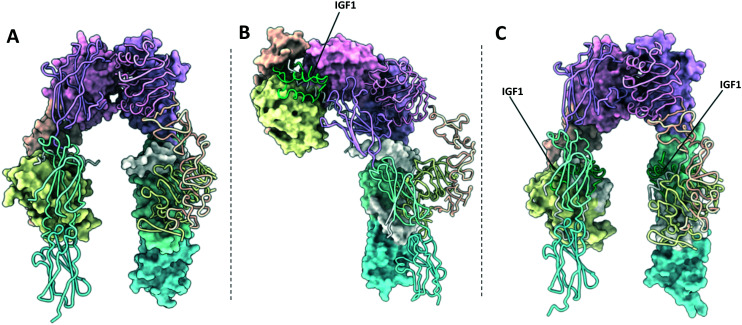
(A) XRCD structure of the apo-IGF1R ectodomain (PDB: 5U8R^3^); (B) crystal structure of the Γ-shaped IGF1R ectodomain bound to a single IGF1 (PDB: 6PYH^5^); (C) crystal structure of the IGF1R ectodomain bound to two IGF1 (PDB: 5U8Q^3^). Note – this structure is unlikely to depict a physiologically relevant conformation of the receptor ectodomain. One monomer in each structure is depicted with a molecular surface and the other in cartoon format. IGF1 is depicted in green.

The activation of IR and IGF1R involves large scale conformational changes in the receptor ectodomain upon ligand binding (Fig. 2 and 3). Assuming that this mechanism is conserved in IR–IGF1R hybrid receptors, the ligand bound conformations of the IR and IGF1R provide regions of the receptors that may be amenable to intervention with small-molecules. Therefore, it is relevant to review the ligand bound structures of the IR and IGF1R, which can be broadly divided into Γ-shaped and T-shaped structures. The following section will briefly summarise the structures of insulin, IGF1 and IGF2, before discussing the available structures of the ligand bound receptors.

### Structures of free insulin, IGF1 and IGF2

**Fig. 4:**
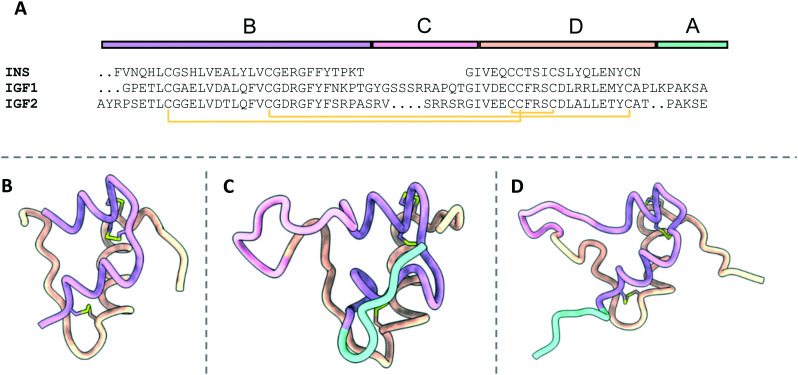
The structures of free insulin, IGF1 and IGF2. B, C, A and D domains are depicted in purple, pink, orange and cyan respectively. Disulfide bonds are depicted in yellow; A) sequence alignment of insulin, IGF1 and IGF2 detailing the locations of their relevant domains; B) the structure of insulin (4INS^13^); C) the structure of IGF1 (2GF1 (ref. 16)); D) the structure of IGF2 (1IGL^19^).

Insulin, IGF1 and IGF2 are highly structurally related, despite each hormone eliciting distinct signalling outcomes (Fig. 4). The free structures of insulin (PDB: 4INS,^13^1HUI,^54^2JV1,^55^2MVC^56^) and IGF1 (PDB: 2GF1,^19^1BQT,^57^1IMX,^58^1HO2 (ref. 59)) have been determined by a combination of XRCD and NMR, whilst the structure of IGF2 is less well studied and has only been determined by NMR (PDB: 1IGL).^19^

The primary difference between the hormones is that IGF1 and IGF2 are composed of a single polypeptide chain, whilst insulin comprises a two-chain polypeptide connected by disulfide bonds. The structure of IGF1 and IGF2 is divided into four domains: B, C, A and D (Fig. 4B and C). Alternatively, insulin is portioned into A and B chains, corresponding to the A and B domains of IGF1 and IGF2 (Fig. 4A).

The primary structural feature of the hormones is three conserved α-helices: two antiparallel α-helices in the A domains connected by a short linker, and a single α-helix in the B domain. The C-domain is a disordered strand which links the B and A chains of IGF1 and IGF2 but is absent in insulin. The short, disordered D-domain extends from the A-domain C-terminus in IGF1 and IGF2. The tertiary structure of the hormones is preserved by three disulfide bonds two interchain disulfides between the A and B domains and a single intrachain disulfide in the A domain.

### Γ-Shaped structure

The single Γ-shaped IR structure is an XRCD structure of an IR ECD construct bound to a single insulin ligand, with separate local refinements in the head and membrane proximal regions giving resolutions of 3.2 Å and 4.2 Å respectfully (PDB: 6HN4 & 6HN5, Fig. 2B).^4^ The structure utilises a leucine zipper fused to the FnIII-3 domains to restore negative cooperativity to the soluble IR ECD, which may have been essential in achieving a singularly bound structure. Low resolution negative-stain electron microscopy images of full-length IRs embedded into lipid nanodiscs support that this conformation is relevant in a membrane bound context.^60^

Comparison of the Γ-shaped structure to the V-shaped apo-structure reveals ligand binding affects large scale conformational rearrangements in the receptor.^4^ The L1/FnIII-2′ interface on the ligand bound monomer is completely disrupted. Consequently, the L1, CR, L2 and αCT′ domains are displaced upwards towards the top of the receptor, whilst the FnIII domains are come together, forming the head region and the perpendicular stalk of the Γ-shape respectively. These rearrangements can be described by two rigid-body rotations, with the L1–CR–L2 domains rotating approximately 35° with respect to the L2–FnIII-1 interdomain linker, and a second a rotation of the L1–CR domains by 55° with respect to the CR–L2 linker.

### T-shaped structures

Several structures detail the IR bound to two or four insulin ligands adopting a T-shaped structure. The first evidence of the IR adopting this topology was found using negative stain EM, imaging full length IR reconstituted into lipid nanodiscs.^60^ Whilst this study did not provide atomic level detail, it confirms the relevance of the V-shaped, Γ-shaped and T-shaped IR structures in a membrane bound context. A subsequent cryo-EM structure shows the IR-ECD bound to two insulin ligands adopting this conformation, which after applying C_2_ symmetry during refinement, achieved a resolution of 4.3 Å (PDB: 6CE9).^61^ Whilst large parts of the FnIII domains are not resolved in this structure, the modelled domains adopt a T-shape, with the ligand binding regions well resolved. Two further cryo-EM structures detail the receptor displaying a T-shaped structure, despite being bound to four insulin ligands. The first structure achieved a global resolution of 4.3 Å (PDB: 6SOF, Fig. 2C),^60^ whilst the second enforced *C*_2_ symmetry to produce a resolution of 3.2 Å (PDB: 6PXW, Fig. 6A).^8^

Analysis of the T-shaped receptor structures show the conformational changes with respect to the apo-receptor are equivalent to those seen in the Γ-shaped structure but occurring in both monomers. Therefore, the head of the T-shaped receptor is comprised of the L1, CR, L2 and αCT domains of both monomers, whilst the FnIII domains associate and extend perpendicular to the centre of the head region.

It is notable that all T-shaped IR structure samples were prepared under supraphysiological insulin concentrations (28 μM,^61^ 40 μM,^14^ 4 : 1 molar ratio insulin: IR^8^). Therefore, it is possible the IR may not bind two or more ligands at physiological insulin concentrations (up to 5 nM (ref. 62)).

## IGF1R ligand bound structures

### Γ-Shaped structure

Six ligand bound structures of IGF1R ectodomain currently exist. Of these, five adopt a Γ-shaped conformation with a single bound ligand. The first Γ-shaped structure is a cryo-EM structure of full-length mouse IGF1R bound to IGF1 at 4.3 Å resolution^5^ (PDB: 6PYH, Fig. 3B). Due to poor resolution of the TMDs and kinase domains, atomic models were only built for the receptor ectodomain. A second cryo-EM structure has been determined of human holo-IGF1R bound to IGF1, albeit to a lower resolution of 7.7 Å.^9,63^ The low resolution of the map prohibited 3D model building of the complex. The same authors also report a cryo-EM structure of human holo-IGF1R bound to insulin at 4.7 Å resolution^63^ (PDB: 6JK8). Once again, limited resolution of the transmembrane and kinase domains meant only the receptor ectodomain and ligand could be modelled. A further two cryo-EM structures detail an IGF1R-ECD construct tethered by a leucine zipper complexed with IGF2.^9^ These two structures are identical in the ligand binding regions but show differing conformations of the FnIII leg regions, such that one structure shows an ‘open legged’ conformation, and the other a ‘closed leg’ conformation. Separate focussed refinements performed on the ‘head’ and ‘leg’ regions for each conformation give four separate atomic models: for the open legged head region (PDB: 6VWG, 3.2 Å), the open legged leg region (PDB: 6VWH, 4.3 Å), the closed legged head region (PDB: 6VWI, 3.7 Å) and the closed legged leg region (PDB: 6VWJ, 4.2 Å).

### T-shaped structures

The final ligand bound structure is an XRCD structure of IGF1R bound to two IGF1 ligands determined to 3.3 Å (PDB: 5U8Q, Fig. 3C).^3^ This structure was produced by soaking the crystals used in the IGF1R apo-structure (5U8R) with IGF1. It appears the constraints of the crystal lattice prevented large scale rearrangement upon the binding of IGF1, with the receptor adopting a conformation intermediate of a V and T-shaped structure. Therefore, whilst this structure doesn't likely represent a physiologically relevant conformation of the IGF1R, it provides a key intermediate conformation as part of a growing portfolio of IGF1R structures.

## Ligand binding

### Site 1

**Fig. 5:**
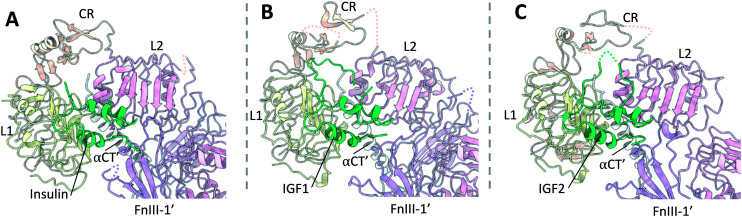
A comparison of insulin, IGF1 and IGF2 bound to site 1 of the IR and IGF1R. (A) Insulin (green) bound to site 1 of the IR (PDB: 6HN5 (ref. 4)); (B) IGF1 (green) bound to site 1 of the IGF1R (PDB: 6PYH^5^); (C) IGF2 (green) bound to site 1 of the IGF1R (PDB: 6VWG^9^).

The ligand bound structures of the IR and IGF1R show that the hormone binding modes are largely conserved between the two receptors (Fig. 5). These structures show that the receptor's primary binding epitope is comprised of residues distributed across the αCT, L1′ and FnIII-1′, which had largely been predicted with prior mutagenesis studies.^64–68^ Insulin, IGF1 and IGF2 bind to these epitopes such that the αCT inserts into a hydrophobic crevice between the A and B chains (Fig. 5A), or A and B domains of IGF1 and IGF2 respectively (Fig. 5B and C). To reveal this hydrophobic cavity, the insulin residues B24–B30 or analogous residues in the IGF1 and IGF2 C-domains detach from the hormone core upon binding to the receptor. Simultaneously, residues on the opposite side of the hormones contact the adjacent loops of the FnIII-1′, such that both monomers of the receptor are contacted simultaneously. This mode of binding had effectively been predicted from kinetic and mutational data in the ‘crosslinking model’.^69^ In line with previous naming conventions, the receptor epitope comprising the αCT and L1′ will hereby be referred to as site 1a, whilst the epitope at the FnIII-1 domain termed site 1b′.

Additionally, IGF1 and IGF2 form contacts to the IGF1R with their C-domains, which extend out into the crevice formed between the L1, CR and L2 domains. This means αCT effectively ‘threads’ the C-domains of the two ligands. Whilst the IGF1 C-domain forms contacts with the IGF1R CR domain, the IGF2 C-domain is four residues shorter and is not long enough to interact with the IGF1R CR domain. Instead, the IGF2 C-domain is stabilised through self-interactions.

### Site 2

**Fig. 6:**
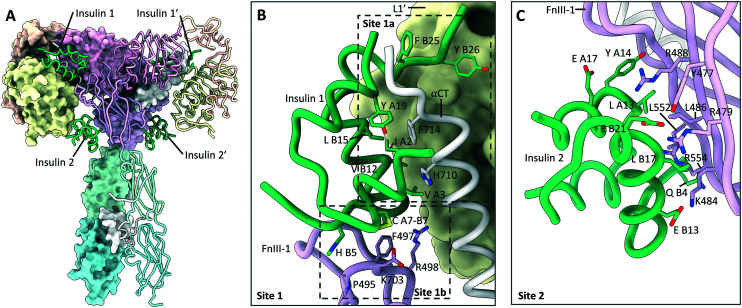
Insulin bound to site 1 and site 2 on the IR. (A) Cryo-EM structure (PDB: 6PXV^8^) of the IR in a T-shaped conformation bound to four insulin (green). Locations of the site 1 and site 2 insulin are highlighted; (B) detailed view (PDB: 6PXV^8^) of insulin (green) bound to the site 1a/1b epitopes, with key residues shown. The locations of the site 1a and 1b epitopes are highlighted; (C) detailed view (PDB: 6PXV^8^) of insulin (green) bound to site 2, with key residues shown.

One of the most notable features of the T-shaped IR structures is the presence of four insulin ligands bound to the receptor.^8,14^ Whilst two of the insulin ligands are bound to the classical 1a/1b′ and 1a′/1b epitopes, two further insulin ligands are bound to symmetry related sites on the FnIII-1 domains. These sites will hereby be referred to as site 2 and 2′ (Fig. 6A and C). Insulin residues that contact the receptor at site 2 are located on the opposite side of the hormone to those contacting site 1. As site 2 is more accessible, it may represent the preliminary insulin binding site, prior to insulin transitioning to the higher affinity site 1. This explains why insulin bound to site 2 is only observed with saturating insulin conditions. Insulin bound to site 2 interact with the L1 domain, and therefore potentially disrupt the L1–FnIII-2′ interface present in the apo-receptor, supporting a model of induced fit for hormone binding.

No analogous site has yet been observed directly for IGF1R. However, the existence of the IGF1R structure bound to two IGF1 ligands^3^ (5U8Q) implies that IGF1 binds to the IGF1R through a mechanism of induced fit. This structure was obtained by crystal soaking of the IGF1R in the apo-conformation, preventing IGF1R from sampling alternative conformations when confined by the crystal lattice. As site 1 is blocked by the FnIII-1 domain in the apo-conformation of the receptors, IGF1 would first have to bind to an accessible site on the receptor, which may represent site 2. Therefore, it seems likely that site 2 is conserved in the IGF1R and hybrid receptors.

### Hybrid ligand binding

The characterisation of site 1a/1a′ and site 1b/1b′ of IR and IGF1R implies that the two primary ligand binding epitopes on a single hybrid receptor are asymmetric *i.e.* do not bind IGF1, IGF2 or insulin with equal affinity. This is because ligands crosslinking sites 1a/1b′ will contact the L1 domain of IR, and the αCT′ and FnIII-1′ of IGF1R or *vice versa*, assuming that this mode of ligand binding is conserved between the IR, IGF1R and the hybrid receptors. This assumption is supported by the single structural model available for the hybrid receptors: a XRCD structure of a hybrid micro receptor construct containing the L1 and CR modules of IR complexed with the αCT region of IGF1R to 3.0 Å.^70^ This structure shows that the hybrid site 1a components assemble analogously to the IR and IGF1R in their apo-conformation. As to which of binding sites has higher affinity for IGF1 in the hybrid receptors, it is difficult to determine without further structural, biochemical or biophysical information.

### Transmembrane domains

**Fig. 7:**
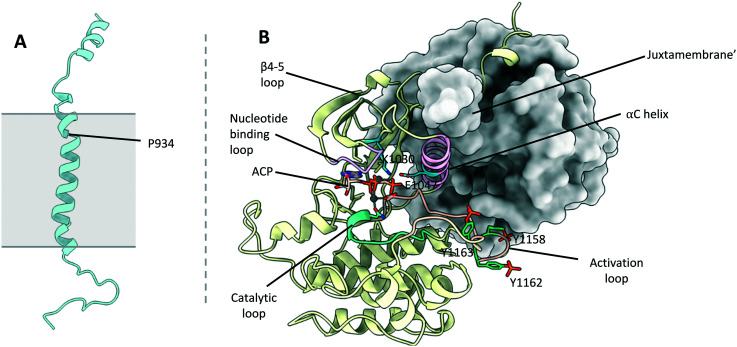
(A) NMR structure of the IR-TMD (PDB: 2MFR^1^). Points of membrane entry are indicated in grey; (B) crystal structure of the IR-TKD activated dimer (PDB: 4XLV^6^). One of the TKDs is depicted in cartoon format and the other with a molecular surface representation. The αC helix (pink), nucleotide binding loop (purple), activation loop (salmon), catalytic loop (cyan) are highlighted. Key residues K1030 and E1047 (blue), aswell as phosphorylation sites Y1158, Y1162 and Y1163 (green) are shown.

The structure of the IR transmembrane region has been shown using NMR to consist of a single α helix in detergent micelles^1^ (PDB: 2MFR, Fig. 7A). This is in line with the structure predicted by molecular dynamics simulations of the IR-TM region in a phospholipid membrane,^71^ indicating that the structure has not been altered through incorporation into lipid vesicles. The α-helix contains a single kink at Gly933, owed to the presence of Pro934 (numbering refers to the IR-B isoform and IGF1R without signal sequence throughout).

Currently no structure exists of the IGF1R TM domains. However, molecular dynamics simulations have modelled the conformation of the TM regions in a phospholipid bilayer.^71^ These simulations show that the IGF1R TM regions adopt a single α-helix with a kink at Pro914, comparable to the IR TMD.

The role of the IR and IGF1R TMD in receptor activation is somewhat debated, although several lines of evidence point to the TMDs dimerising during receptor activation.^39^ Whilst the IR-TMDs were found to be monomeric in detergent micelles, crosslinking studies indicated that they could dimerise within the detergent micelles depending on the protein/detergent ratio.^1^ Detergent micelles are relatively poor mimics of the bilayer and may hinder dimerization. This is supported by NMR experiments of isolated IR-TMDs, showing they can adopt dimeric conformations in membrane-like environments.^72^ Similarly, molecular dynamics simulations and FRET analysis indicate that the IGF1R-TMD regions dimerise upon IGF1R activation.^73^ This work suggests that the TM regions associate near the kink at Pro914.

Conflicting evidence arises for the IR-TMD, with experiments showing that the IR can be activated by a 24-residue peptide corresponding to the IR-TMD.^74^ The authors propose the IR-TMD are dimerised in the basal state, and separated in the activated receptor, hence the IR-TMD peptide can intercalate and disrupt the basal state dimer to result in activation of the receptor. However, there is no additional structural data to support this mechanism. Additionally, the very existence of functional IR–IGF1R hybrid receptors implies the mechanism of TMD activation must be conserved between the IR and IGF1R. Therefore, it seems likely that the TMDs of the IR–IGF1R hybrids dimerise during the activation of the hybrid receptors. This raises the possibility of designing small molecule mimetics of the IR and IGF1R TMDs, which could potentially disrupt the dimerization of the TMD helix during signal transduction across the membrane.

## Kinase domain structures

### IR kinase domain

**Fig. 8:**
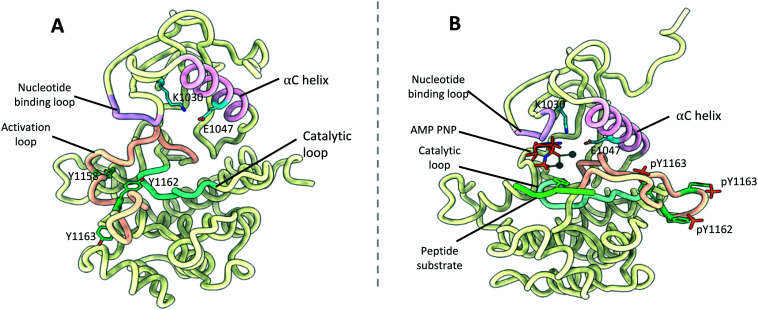
(A) Crystal structure of the inactivated IR kinase domain (PDB: 1IRK^7^) with the αC helix (pink), nucleotide binding loop (purple), activation loop (salmon), catalytic loop (cyan) highlighted. Key residues K1030 and E1047 (blue), aswell as phosphorylation sites Y1158, Y1162 and Y1163 (green) are shown; (B) crystal structure of the phosphorylated (activated) IR kinase domain (PDB: 1IR3 (ref. 10)) with the αC helix (pink), nucleotide binding loop (purple), activation loop (salmon), catalytic loop (cyan) and bound peptide substrate (lime) highlighted. Key residues K1030 and E1047 (blue), aswell as phosphorylated residues pY1158, pY1162 and pY1163 (green) are shown.

Structures for the inactive and phosphorylated (active) states of the IR TKD have been determined by XRCD to 2.1 Å (PDB: 1IRK, Fig. 8A)^75^ and 1.9 Å (PDB: 1IR3, Fig. 8B) respectively.^10^ The latter is complexed with a peptide substrate and ATP analogue. Similarly, a structure of the IGF1R TKD in its active state (PDB: 1K3A)^76^ has been determined to 2.1 Å, whilst two structures of the inactive state (PDB: 1M7N,^77^1P40 (ref. 78)) have been elucidated to 2.7 Å and 1.5 Å respectively. Several structures of the IR and IGF1R kinase domains bound to small-molecule inhibitors have since been published.^79–84^

The IR and IGF1R TKDs show typical kinase topology; comprised of a small N-terminal and larger C-terminal lobe connected by a single linker. The C-terminal lobe encompasses the active site and activation loop, the latter containing three IR tyrosine phosphorylation sites Tyr1158, Tyr1162 and Tyr1163 (Tyr1134, Tyr1138 and Tyr1139 in IGF1R).^76,85,86^ The activation loop itself traverses the catalytic cleft between the two lobes. In the basal state, Tyr1162 and Tyr1138 occupy their respective receptors active site, causing the activation loop blocks access to the ATP binding site. This prevents *cis* phosphorylation of the catalytic tyrosine residues in the basal state conformation (Fig. 8A).

Upon receptor activation, the TKDs effect *trans*-autophosphorylation of the three catalytic tyrosine residues. To stabilise the phosphorylated tyrosine groups, the activation loop is significantly displaced from its basal state conformation. This provides unrestricted access to the kinase active site and ATP binding site (Fig. 8B).

Additionally, the TKDs from each monomer of the IR and IGF1R associate in the active state, functioning as a dimeric unit to promote optimal substrate phosphorylation and subsequent downstream signalling. This has been shown with a crystal structure of the phosphorylated IR TKDs and preceding juxta membrane region to 2.3 Å (PDB: 4XLV), in which the TKDs adopt a dimeric conformation (Fig. 7B). Subsequent biochemical studies determined the IGF1R TKD forms a similar activated dimer.^6^ In the structure, the juxta membrane region binds to a cavity formed between the β-sheet and α-helix C (αC). The αC helix is known to be an important regulatory element in protein kinases, typically forming a glutamate–lysine ion pair which coordinates the α and β phosphates of ATP in the active kinase.^87^ The dimeric conformation of the IR and IGF1R kinase domains promotes the formation the salt bridge between Lys1030 and Glu1047 (Lys1006 and Glu1023 in IGF1R respectively), which in turn stimulates binding of ATP and substrate phosphorylation. The formation of an activated tyrosine kinase dimer in both the IR and IGF1R raises the possibility of a similar mechanism occurring in the IR–IGF1R hybrid receptors. If the tyrosine kinases formed an active dimer with a similar conformation in the hybrid receptors, it raises the possibility of utilising small molecules which bind to the β-sheet αC helix cleft to prevent the formation of the activated dimer and inhibit hybrid signalling.

## Small molecule modulators of the IR and IGF1R

**Fig. 9:**
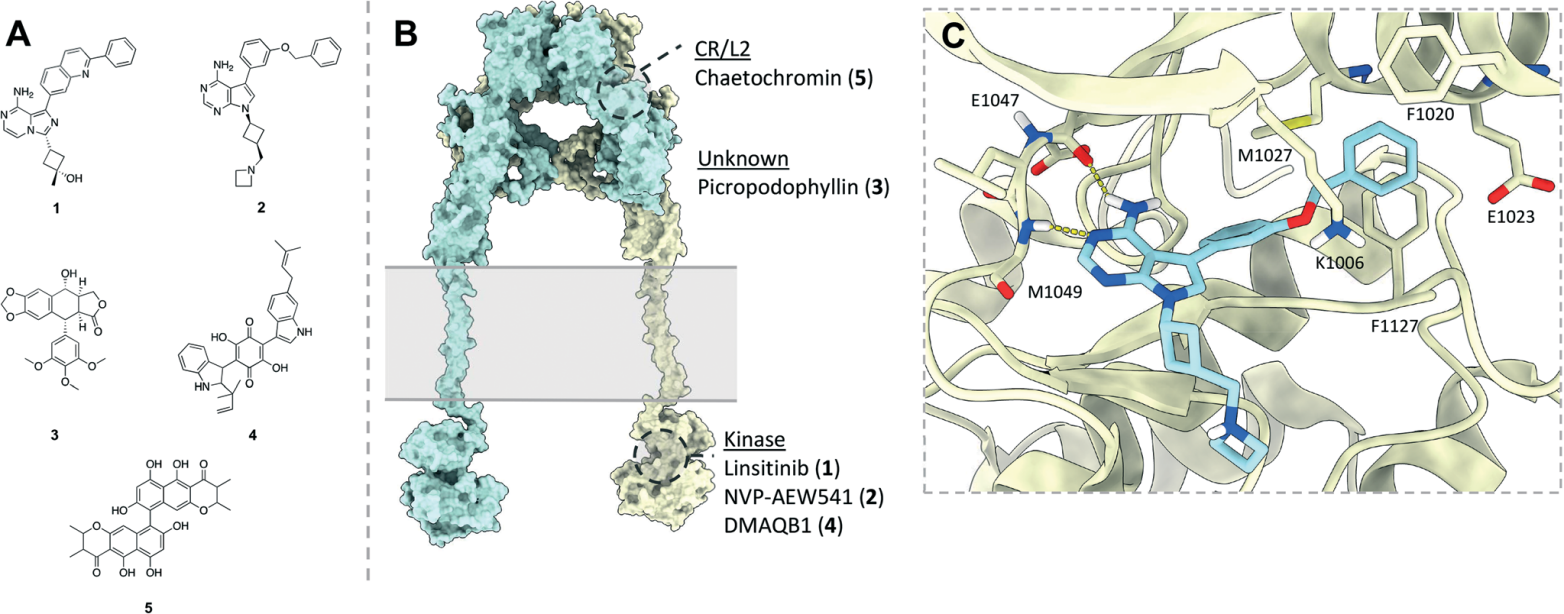
(A) Structures of selected small molecules which modulate the IR and/or IGF1R; (B) representative model of the full length IR/IGF1R (produced from PDB: 4ZXB,^2^2MFR,^1^1IRK^7^) indicating the regions which selected small molecule IR/IGF1FR modulators are believed to bind. Point of membrane entry for the receptors is denoted in grey. Of these molecules, only NVP-AEW541 has a published protein co-ligand structure confirming its binding mode. Note – linsitinib (1), NVP-AEW541 (2) and picropodophyllin (3) are presented here as inhibitors of the IGF1R, whilst DMAQB1 (4) and chaetochromin (5) are presented as activating the IR; these are only displayed on a single model of an IR/IGF1R receptor for illustrative purposes; (C) XRCD structure (PDB: 5HZN) of NVP-AEW541 (blue) bound to the IGF1R kinase (yellow) detailing key binding interactions.

The following section will briefly describe some of notable classes of small molecule modulators effecting the IR and IGF1R, to allow discussion of the relevance of these for modulating IR–IGF1R hybrid function. Efforts to develop small molecule modulators the IR and IGF1R have focussed on identifying IGF1R antagonists and IR agonists (Fig. 9A and B), owed to the receptors' respective roles cancer and diabetes.

### IGF1R ATP competitive inhibitors

The search for IGF1R antagonists has largely focussed on developing ATP competitive kinase inhibitors. Due to the high sequence homology between the IR and IGF1R ATP binding pocket achieving selectivity is challenging. This is illustrated by linsitinib (1), a dual IGF1R/IR kinase inhibitor with IC_50_ values of 35 nM and 75 nM respectively.^88^*In vivo* analysis determined that linsitinib showed a dose-dependent effect on tumour growth inhibition.^88^ However, linsitinib did not progress past phase 3 clinical trials for metastatic cancer due to lack of efficacy. Patients also experienced side effects which could be attributed to disruption of insulin signalling such as hyperglycaemia.^89^

Additional IR/IGF1R tyrosine kinase inhibitors showing improved selectivity profiles have been developed, such as NVP-AEW541 developed by Novartis (2).^90^ This compound has cellular IC_50_ values of 0.086 μM and 2.3 μM for the IGF1R and IR respectively,^90^ and similarly reduced the growth of tumour xenografts *in vivo*.^91^ An X-ray co-crystal structure of NVP-AEW541 bound to the IGF1R kinase domain (PDB: 5HZN) has been elucidated to 2.20 Å, determining that the molecule forms hydrogen bonds with the canonical ATP binding hinge of the kinase, and disrupts formation of the salt bridge between Lys1033 and Lys1055 present in the activated kinase (Fig. 9C).^80^ This causes movement of the αC helix, opening a lipophilic pocket into which the benzyloxy moiety of NVP-AEW541 can insert to confer high selectivity over the IR. However, NVP-AEW541 suffered from lack of efficacy and acquired resistance when tested against breast cancer cell lines.^92^ Challenges in targeting the IGF1R with ATP competitive inhibitors in oncology largely centre around lack of efficacy due to compensatory signalling from the other growth factors (including IR and hybrids), and side effects associated with inhibition of the IR.^93^ It follows that ATP competitive inhibitors would not be suitable for targeting the IR–IGF1R hybrid receptors, as these would simultaneously disrupt IR and IGF1R homodimer signalling.

### Picropodophyllin

The cyclolignan picropodophyllin (3) (PPP) is an allosteric IGF1R inhibitor. PPP was originally tested as an IGF1R inhibitor as it mimics the conformation of the IGF1R residues 1135 and 1136, and was therefore predicted to interfere with IGF1R at the substrate level. PPP reduces IGF1R phosphorylation in immunoprecipitation experiments.^94^ Notably, PPP was found to have no effect on the basal phosphorylation of IR.^94^ There is evidence that PPP additionally induces degradation of IGF1R, with an approximate 30–50% reduction in IGF1R after 12 hour of treatment in cultured cells,^95^ and may be key to the potent antitumour effects of PPP. PPP induced IGF1R degradation appears dependent on the MDM2 E3 ligase, as altered expression was abrogated in cells expressing truncated MDM2 lacking the ligase domain.^95^ AXL1717, a bio-orally available analogue of PPP has since entered phase 2 clinical trials for treatment of non-small cell lung cancer, although it was found to provide no benefit for progression or overall survival.^96^

Downregulation of the IGF1R with PPP analogues represents an alternative strategy for inhibiting IR–IGF1R hybrid signalling; genetic manipulation of IGF1R expression has previously been utilised to alter hybrid receptors populations in mice.^40,41^ However, this implicitly alters IGF1R signalling, meaning such compounds are of limited use as tools to elucidate the signalling capabilities of the IR–IGF1R hybrids. Therefore, compounds downregulating IGF1R offer no advantage over the current methods available to study IR–IGF1R hybrids.

### Insulin mimetics

Finally, several molecules representing a class of non-peptidic IR agonists have been identified. Such compounds are of particular interest as small-molecule insulin mimetics may provide an orally available insulin alternative. These first of these is demethylasterriquinone (DMAQ) B1 (4), which has been shown to directly induce IR kinase phosphorylation, resulting in a reduction of blood glucose in mice.^97^ Several lines of evidence point to this compound acting on the tyrosine kinase domain, although this has not been shown definitively.^97^ Whilst DMAQB1 shows good oral bioavailability, it is only moderately selective and highly cytotoxic.

Several derivatives of DMAQB1 have been investigated as IR agonists. Most recently, the insulin mimetic chaetochromin (5) has been determined to activate IR, whilst showing improvement in selectivity and cytotoxicity relative to DMAQB1.^98^ Flexible docking of chaetochromin to the insulin receptor apo-ectodomain structure suggested it binds at hinge region between the CR and L2 domains of the IR. Additional partial proteolysis and HDX-coupled mass spectrometry confirmed that the molecule bound to the receptor ectodomain. However, there is no further evidence on how DMAQB1 and chaetochromin activate the IR. Further mechanistic studies are necessary to determine if this class of insulin mimetics are suitable for targeting IR–IGF1R hybrids.

## Conclusions and future outlook

A single receptor coupling nutrition to cellular growth and metabolism remains important in less complex animals. During evolution this single receptor developed into a complex system of cell surface receptors specialized to regulate metabolism (IR) and growth (IGF1R). A third receptor, the IR–IGF1R hybrid, is expressed as abundantly as IR and IGF1R but unlike IR and IGF1R the role of hybrids has remained a mystery. The association of IR–IGF1R hybrid receptors with insulin resistance and cancer drug resistance means they provide promising novel target in metabolic disease and oncology. Furthermore, developing selective inhibitors of the IR–IGF1R hybrid signalling would allow better understanding of receptors role in human physiology, through differentiating homodimeric and heterodimeric IR–IGF1R signalling. The major challenge in modulating hybrid function is achieving selectivity over the IR and IGF1R. Current modulators of the IR and IGF1R will naturally act against the homodimeric receptors and are therefore not suitable for repurposing as modulators of IR–IGF1R hybrids. To elicit selectivity, it is necessary to target the regions of lowest structure conservation between the homodimeric and heterodimeric receptors. It follows targeting interfaces formed between each receptor monomer in the hybrid receptor is most likely to provide selectivity, as these interfaces are not conserved in the IR and IGF1R homodimers. To disrupt the receptor function, these interfaces must either be present in the apo-receptor to disrupt heterodimer formation pre-disulfide linkage or be essential for the robust activation of the receptor upon ligand binding.

Structural information from the IR and IGF1R homodimers suggests the following regions of the hybrid receptors as promising targets 1) the interfaces formed between monomers in the apo-receptor ectodomain: the L1–FnIII-2′ and L2–FnIII-1′ interfaces. If either of these interactions could be disrupted be prior to disulfide formation, it may prevent hybrid formation 2) The 1a/1b′ ligand binding pocket formed of the αCT, L1′ and FnIII-1. Binding of small molecules here may disrupt ligand binding. 3) The interface formed between the transmembrane domains during receptor activation. TMD dimerization appears to be a key event in IR and IGF1R activation. Interfering with this could prevent the activation of the cytoplasmic kinase domains. 4) Preventing dimerization of the tyrosine kinase domains. The activated dimer formed during IR and IGF1R activation promotes downstream signalling. Therefore, disrupting the association of the TKDs may inhibit downstream signalling.

Information to facilitate structure-based design of small molecules targeting these interfaces would greatly augment the search for hybrid modulators. Clearly, an atomic resolution structure for the hybrids would be indispensable for the rationale design of small molecules targeting these interfaces. However, the hybrid receptors remain a formidable structural challenge due to their membrane bound nature, structural flexibility and difficulties associated with purifying them from IR and IGF1R homodimers. Complementary mutagenesis could be used to validate the importance of the above interfaces in hybrid formation and function, as well as allowing targeting of specific hotspot residues. In conclusion, whilst there remain several challenges in developing small-molecules modulators of IR–IGF1R hybrid formation, there is a clear opportunity to target these receptors as for the treatment for type 2 diabetes and/or certain cancers.

## Author contributions

SJT wrote the initial manuscript. KJS, SJT and MJM prepared the manuscript. All authors analysed and discussed the contents of the review.

## Conflicts of interest

The authors declare no conflicts of interest.

## Supplementary Material

MD-013-D1MD00300C-s001
