# Supplementary material for: Recent developments in the structural characterisation of the IR and IGF1R: implications for the design of IR–IGF1R hybrid receptor modulators
Source: RSC Med Chem. 2022 Feb 21;13(4):360–74. doi: 10.1039/d1md00300c (PMC9020618; doi:10.1039/d1md00300c)
Supplement: MD-013-D1MD00300C-s001 [file MD-013-D1MD00300C-s001.pdf]

Electronic Supplementary Material (ESI) for RSC Medicinal Chemistry.
This journal is © The Royal Society of Chemistry 2022

Supplementary figures

Table S1: A summary of all the current IR, IGF1R and hybrid receptor structures. Small molecule bound kinase structures have been omitted for clarity.

Receptor	Construct	Technique	Fab	Ligand	Resolution (Å)	PDB	Date
IR	TK domain (apo)	XRCD	None	-	2.1	1IRK	1994(1)
IR	TK domain (activated)	XRCD	None	-	1.9	1IR3	1997(2)
IR	L1-CR-L2	XRCD	None	None	2.3	2HR7	2006(3)
IR	ECD homodimer	XRCD	2 x (83-7 + 83-14)	None	3.8	2DTG	2006(4)
					3.8	3LOH	2010(5)
IR	L1-CR + αCT$^{704-719}$	XRCD	83-7	Ins	3.9	3W11	2013(6)
					3.3	4OGA	2014(7)
IR	[L1-CR-L2-(FnIII-I)-αCT$^{704-719}$]$_2$	XRCD	2 x 83-14	Ins	4.4	3W14	2013(6)
IR	L1-CR + αCT$^{697-719}$	XRCD	83-7	[D-Pro^{B26}]-DTI-NH_2	4.3	3W13	2013(6)
IR	TM domain	NMR	None	-	-	2MFR	2014(8)
IR	L1-CR-αCT$^{697-719}$	XRCD	None	None	3.0	4XST	2015(9)
IR	ECD homodimer	XRCD	2 x (83-7 + 83-14)	None	3.3	4ZXB	2016(10)
IR	ECD homodimer	EM	None	2 x Ins	4.3	6CE9	2018(11)
				1 x Ins	7.4	6CE7	
IR	ECD homodimer (w / leucine zipper)	EM	None	1 x Ins	3.2	6HN5	2018(12)
					4.2	6HN4	
IR	Full length (Only ECD modelled)	EM	None	4 x Ins	3.2	6PXV	2019(13)
					3.2	6PXW	
IR	ECD homodimer	EM	None	4 x Ins	4.3	6SOF	2020(14)
IGF1R	TKD (activated)	XRCD	None	-	2.1	1K3A	2001(15)
IGF1R	TKD (apo)	XRCD	None	-	1.5	1P4O	2003(16)

IGF1R	L1-CR-L2	XRCD	None	None	2.6	1IGR	1998(17)
IGF1R	ECD Homodimer	XRCD	2 x 24-60	None	3.0	5U8R	2018(18)
				2 x IGF1	3.3	5U8Q	
IGF1R	Full length	EM	None	IGF1	4.3	6PYH	2019(19)
IGF1R	ECD homodimer	EM	None	Ins	4.7	6JK8	2020(20)
				IGF1	7.7	EMD-0741	
IGF1R	Full length (Leucine zipper)	EM	None	IGF2	3.2	6VWG	2020(21)
					4.3	6VWH	
					3.7	6VWI	
					4.2	6VWJ	
Hybrid	IR: L1-CR + IGF1R: $\alpha CT^{691-706}$	XRCD	None	IGF1	3.0	4XSS	2015(9)

Supplemental References

1. Stevan RH, Lei W, Wayne AH. Crystal structure of the tyrosine kinase domain of the human insulin receptor. Nature. 1994;372(6508):746.
2. Hubbard SR. Crystal structure of the activated insulin receptor tyrosine kinase in complex with peptide substrate and ATP analog. The EMBO journal. 1997;16(18):5572-81.
3. Meizhen L, Thomas PJG, Neil MM, Peter AH, Epa VC, John DB, et al. The first three domains of the insulin receptor differ structurally from the insulin-like growth factor 1 receptor in the regions governing ligand specificity. Proceedings of the National Academy of Sciences. 2006;103(33):12429.
4. McKern NM, Lawrence MC, Streltsov VA, Lou MZ, Adams TE, Lovrecz GO, et al. Structure of the insulin receptor ectodomain reveals a folded-over conformation. Nature. 2006;443(7108):218-21.
5. Smith BJ, Huang K, Kong G, Chan SJ, Nakagawa S, Menting JG, et al. Structural resolution of a tandem hormone-binding element in the insulin receptor and its implications for design of peptide agonists. Proc Natl Acad Sci U S A. 2010;107(15):6771-6.
6. Menting JG, Whittaker J, Margetts MB, Whittaker LJ, Kong GK, Smith BJ, et al. How insulin engages its primary binding site on the insulin receptor. Nature. 2013;493(7431):241-5.
7. Menting JG, Yang Y, Chan SJ, Phillips NB, Smith BJ, Whittaker J, et al. Protective hinge in insulin opens to enable its receptor engagement. Proc Natl Acad Sci U S A. 2014;111(33):E3395-404.
8. Li Q, Wong YL, Kang C. Solution structure of the transmembrane domain of the insulin receptor in detergent micelles. Biochim Biophys Acta. 2014;1838(5):1313-21.
9. John, Callum, Geoffrey, Mai, Colin, Michael. Structural Congruency of Ligand Binding to the Insulin and Insulin/Type 1 Insulin-like Growth Factor Hybrid Receptors. Structure. 2015;23(7):1271-82.
10. Croll TI, Smith BJ, Margetts MB, Whittaker J, Weiss MA, Ward CW, et al. Higher-Resolution Structure of the Human Insulin Receptor Ectodomain: Multi-Modal Inclusion of the Insert Domain. Structure. 2016;24(3):469-76.
11. Scapin G, Dandey VP, Zhang Z, Prosise W, Hruza A, Kelly T, et al. Structure of the insulin receptor-insulin complex by single-particle cryo-EM analysis. Nature. 2018;556(7699):122-5.
12. Weis F, Menting JG, Margetts MB, Chan SJ, Xu Y, Tennagels N, et al. The signalling conformation of the insulin receptor ectodomain. Nat Commun. 2018;9(1):4420.
13. Uchikawa E, Choi E, Shang GJ, Yu HT, Bai XC. Activation mechanism of the insulin receptor revealed by cryo-EM structure of the fully liganded receptor-ligand complex. Elife. 2019;8.
14. Gutmann T, Schafer IB, Poojari C, Brankatschk B, Vattulainen I, Strauss M, et al. Cryo-EM structure of the complete and ligand-saturated insulin receptor ectodomain. J Cell Biol. 2020;219(1).
15. Favelyukis S, Till JH, Hubbard SR, Miller WT. Structure and autoregulation of the insulin-like growth factor 1 receptor kinase. Nature Structural Biology. 2001;8(12):1058-63.
16. Munshi S, Hall DL, Kornienko M, Darke PL, Kuo LC. Structure of apo, unactivated insulin-like growth factor-1 receptor kinase at 1.5 Å resolution. Acta Crystallographica Section D. 2003;59(10):1725-30.

17. Thomas PJG, Neil MM, Meizhen L, Maurice JF, John DB, George OL, et al. Crystal structure of the first three domains of the type-1 insulin-like growth factor receptor. Nature. 1998;394(6691):395.
18. Xu Y, Kong GK, Menting JG, Margetts MB, Delaine CA, Jenkin LM, et al. How ligand binds to the type 1 insulin-like growth factor receptor. Nat Commun. 2018;9(1):821.
19. Li J, Choi E, Yu H, Bai XC. Structural basis of the activation of type 1 insulin-like growth factor receptor. Nat Commun. 2019;10(1):4567.
20. Zhang X, Yu D, Sun J, Wu Y, Gong J, Li X, et al. Visualization of Ligand-Bound Ectodomain Assembly in the Full-Length Human IGF-1 Receptor by Cryo-EM Single-Particle Analysis. Structure. 2020;28(5):555-61 e4.
21. Xu Y, Kirk NS, Venugopal H, Margetts MB, Croll TI, Sandow JJ, et al. How IGF-II Binds to the Human Type 1 Insulin-like Growth Factor Receptor. Structure. 2020.
